# Growing recognition of transgender health

**DOI:** 10.2471/BLT.16.021116

**Published:** 2016-11-01

## Abstract

Stigma, discrimination and lack of legal recognition remain major barriers for transgender people to access the health services they need. Vijay Shankar Balakrishnan reports.

Olga Aaron was registered male at birth, but always felt that she was a woman. Following a mental health assessment and with the support of her family, at 18 years Aaron underwent surgery and hormone therapy (and a name change) to become a woman.

Two decades later, Aaron is campaigning for the social acceptance and recognition of transgender women, like herself, and to raise awareness about their specific health issues in India.

“Transgender women are marginalized and denied basic human rights,” says Aaron, who lives in the city of Chennai.

“Transgender women are marginalized and denied basic human rights.”

Olga Aaron

“I was lucky to have an understanding family who supported me while I studied at university,” says Aaron, who managed to obtain a bachelor’s degree in English literature and a master’s degree in public administration from Madras University by distance learning. 

Aaron is one of an estimated 25 million people, or 0.3 to 0.5% of the global population, who are transgender. This first global estimate of their numbers was published in a study in the *Lancet* in June.

“Transgender is an umbrella term for people whose gender identity is different from the sex they were assigned at birth,” says Annette Verster, a technical officer in the HIV Department at the World Health Organization (WHO) in Geneva.

A person’s gender identity is their own sense of whether they are male or female, or neither. Some transgender people identify themselves with their changed gender: from male to female or female to male. Others see themselves as members of a third sex. 

In Thailand transgender people are known as *kathoey*, in Indonesia *waria*, in Mexico *muxe* and**in Bangladesh and Pakistan *hijra, *(in India the term refers to transgender women). 

“Identity documents that do not match a person’s gender can hinder access to health services, social protection and employment,” Verster says. “Transgender women may also be subject to punitive laws and discriminatory policies affecting men who have sex with men.”

WHO’s Member States have committed themselves to providing universal coverage of health services in their efforts to achieve the sustainable development goals by 2030. “Reaching marginalized groups, such as transgender people, will be essential,” Verster says.

Transgender people are one of five groups that are disproportionately affected by HIV globally, according to WHO’s *Guidelines on HIV prevention, diagnosis, treatment and care for key populations *released in 2014. The others are: people who inject drugs, men who have sex with men, sex workers and prisoners.

“These groups are defined as key populations for the HIV response because they are at increased risk of HIV infection. In addition, they are often marginalized, stigmatized and criminalized which affects their ability to access health services, including HIV prevention, testing and treatment,” Verster says.

“It is estimated that in low- and middle-income countries transgender women are around 49 times more likely to be living with HIV****than other adults of reproductive age,” says Verster, citing a 2013 study published in *The Lancet* *infectious diseases –* the first to estimate the HIV burden among transgender women outside the United States of America (USA).

The authors found that data on HIV prevalence in transgender women were only available for 15 countries and, of those, India had the highest prevalence with 43.7% of the 135 study participants living with HIV.

Transgender people in India are considered to be of low status according to Hindu mythology. “Many transgender women are rejected by their families in India. Even if they have an education, they struggle to find employment and often end up as sex workers and beggars,” Aaron says.

Following the WHO key populations guideline released in 2014, WHO published a technical brief with its partners, in 2015 entitled *HIV and young transgender people* on how best to provide health services, programmes and support for young transgender people. In the same year a WHO Policy Brief on *Transgender people and HIV* was published summarizing relevant WHO recommendations.

In addition, WHO and its partners developed a guide on how to use the technical guidance, entitled *Implementing comprehensive HIV and STI programmes with transgender people,* which was published this year by the United Nations Development Programme. 

While preparing the WHO guidance, Verster and her colleagues did a qualitative survey with transgender people from around the world.

“We found that transgender people tend to have health priorities other than HIV,” Verster says, adding: "Unless health services are designed in accordance with the needs of transgender people and in consultation with them, it may be difficult to reach them with HIV prevention and care.”

“Unless health services are designed in accordance with the needs of transgender people and in consultation with them, it may be difficult to reach them with HIV prevention and care.”

Annette Verster

Not all transgender people seek gender affirming treatment. For those who do, hormone therapy is the main medical intervention to acquire sex characteristics aligned with the individual's gender identity, according to the Center of Excellence for Transgender Health at the University of California in San Francisco. 

Transgender people may seek a range of gender affirming surgeries, including procedures that are also performed in non-transgender populations.

“Not everyone who wants gender affirming surgery in India can access it. Some transgender people remove their genitals by self-mutilation, others go to ‘quacks’,” says Aaron, who campaigns to raise awareness about transgender issues in India.

“HIV is not the only health issue that transgender people face,” says Aaron, adding that they face mental health issues including depression, mood and anxiety disorders and suicidal ideation. Studies from the Islamic Republic of Iran and Nepal highlight the prevalence of mental health issues in transgender people.

Dr Sari Reisner, an epidemiologist at the Boston Children's Hospital in the USA and a transgender man himself, agrees that there are many health issues that transgender people face other than HIV infection, but that there is very little research on these other health issues.

Indeed, this is what Reisner and his fellow researchers discovered when they reviewed the scientific literature on transgender people’s health over the last five years. 

In their findings published in a *Lancet* series on the subject in June, Reisner and his colleagues found that most health research divides the human population into male and female, although recently studies started to include a third category for transgender people. 

Most of the health data they found on transgender people were on HIV infection, mental health, sexual and reproductive health, substance use disorders, violence and victimization, and on the effects of stigma and discrimination such as mental health issues. 

The data revealed major health inequities between transgender people and many other members of society and that transgender people were often unable to access the health services they need because of their social and economic marginalization.

Reisner and his colleagues proposed four ways forward: count the transgender population globally; address stigma and other issues that make transgender people vulnerable to health risks; recognize their human and legal rights so that they can be covered by health services; and do health research *with* transgender people rather than *on* them.

Several countries – including Argentina, Australia, Bangladesh, India, Nepal, New Zealand, Pakistan, Portugal and Thailand – have legislation recognizing the rights of transgender people in some way.

In India, the government started offering free gender affirming surgery in 2009, although this is still not accessible everywhere in the vast country of 1.2 billion people. 

In Argentina, a 2012 law – the first of its kind according to Reisner – allows transgender people to change their legal gender identity from male to female or vice versa through a simple administrative process.

In Canada, gender affirmation surgery and treatment are covered by the publicly-funded health insurance system and some universities are integrating transgender health into the medical curricula.

“There is a broader acceptance and a desire on the part of health-care providers to learn about lesbian, gay, bisexual, transgender and queer (LGBTQ) health issues,” says Dr Ed Kucharski, who is responsible for incorporating these issues into the curriculum at the University of Toronto.

“Few textbooks include LGBTQ health issues, so we use materials from LGBTQ community centres and from the web,” says Kucharski.

The university is developing traditional and online content that medical students can use to learn how to care appropriately for transgender patients. Kucharski explains: “In the next year or so we will be developing a virtual patient that students can interact with.”

Another key development in gaining wider recognition for transgender health issues is the revision of the *International statistical classification of diseases* *and related health problems *(ICD), the standard diagnostic reference book for epidemiology, health management and clinical practice.

In the original version of the current edition, ICD-10, “gender identity disorders” were classified under “mental and behavioural disorders”. But in the next edition, ICD–11, which was released for Member State comments last month and is due to be published in 2018, transgender health issues appear in a new category of “gender incongruence”.

For Reisner this classification shift reflects the struggle for transgender health issues to find their place. “There are many opinions from many sides on this,” Reisner says, welcoming the shift: “I don’t think transgender issues need to be seen as a mental disorder.”

**Figure:**
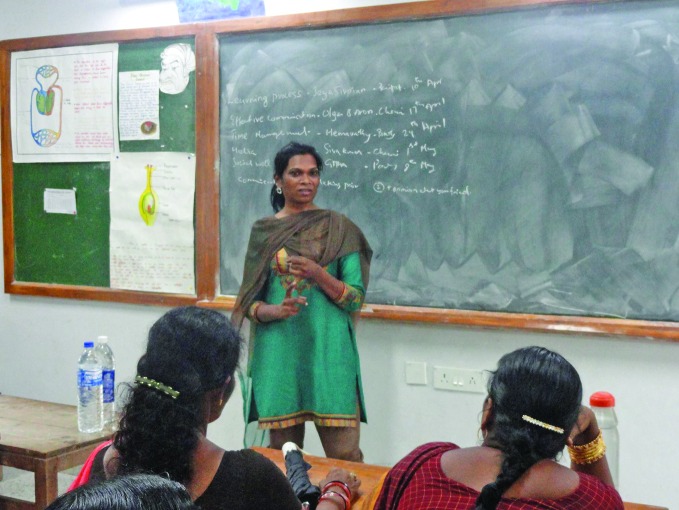
Olga Aaron providing education and raising awareness about transgender issues in India

Courtesy of Olga Aaron

